# Contrasting behavior between two populations of an ice‐obligate predator in East Antarctica

**DOI:** 10.1002/ece3.2652

**Published:** 2016-12-20

**Authors:** Karine Heerah, Mark Hindell, Virginia Andrew‐Goff, Iain Field, Clive R. McMahon, Jean‐Benoît Charrassin

**Affiliations:** ^1^ LOCEAN LaboratorySorbonne Universités (UPMC, Univ Paris 06)‐CNRS‐IRD‐MNHNParisFrance; ^2^ Institute for Marine and Antarctic StudiesUniversity of TasmaniaHobartTas.Australia; ^3^ Antarctic Climate and Ecosystem Cooperative Research CentreUniversity of TasmaniaHobartTas.Australia; ^4^ Department of Biological SciencesMacquarie UniversitySydneyNSWAustralia; ^5^ Sydney Institute of Marine ScienceSydneyNSWAustralia

**Keywords:** capital breeder, first‐passage time, habitat use, movement patterns, pinnipeds, polar regions, winter

## Abstract

The Austral autumn–winter is a critical period for capital breeders such as Weddell seals that must optimize resource acquisition and storage to provision breeding in the subsequent spring. However, how Weddell seals find food in the winter months remains poorly documented. We equipped adult Weddell seals after their annual molt with satellite‐relayed data loggers at two sites in East Antarctica: Dumont D'Urville (*n* = 12, DDU) and Davis (*n* = 20). We used binomial generalized mixed‐effect models to investigate Weddell seals’ behavioral response (i.e., “hunting” vs. “transit”) to physical aspects of their environment (e.g., ice concentration). Weddell seal foraging was concentrated to within 5 km of a breathing hole, and they appear to move between holes as local food is depleted. There were regional differences in behavior so that seals at Davis traveled greater distances (three times more) and spent less time in hunting mode (half the time) than seals at DDU. Despite these differences, hunting dives at both locations were pelagic, concentrated in areas of high ice concentration, and over areas of complex bathymetry. There was also a seasonal change in diving behavior from transiting early in the season to more hunting during winter. Our observations suggest that Weddell seal foraging behavior is plastic and that they respond behaviorally to changes in their environment to maximize food acquisition and storage. Such plasticity is a hallmark of animals that live in very dynamic environments such as the high Antarctic where resources are unpredictable.

## 1 Introduction

Individuals that optimize resource acquisition are expected to increase their chances of reproductive success and survival, thereby increasing their fitness (Stearns, 1992). In the case of marine predators that must also contend with their prey being vertically distributed in the water column as well as geographically, this can be achieved by decreasing displacement speed and increasing the sinuosity of their trajectory through the water column both in the horizontal and in vertical dimensions (Kareiva & Odell, 1987). This behavior called area‐restricted search (ARS) is commonly used to detect foraging activity and is contrasted with transit behavior during which the animals travel faster and more linearly (Fauchald & Tveraa, 2003). Detecting these behavioral changes (i.e., between transiting and ARS) and quantifying the relationships between animal behavior and the associated environmental features are crucial to understanding predators’ fitness and survival (Bestley, Jonsen, Hindell, Harcourt, & Gales, 2015). This is particularly important for high‐latitude animals given the restrictions their environment imposes on their foraging behavior and the importance of storing food for the short, but energetically demanding breeding season.

Throughout winter, high‐latitude predators face increased sea‐ice cover and modified hydrological regimes, as well as lower marine productivity due to limited sunlight and nutrient input (Tynan, Ainley, & Stirling, 2009). In Antarctica, the sea‐ice on the continental shelf is a key overwinter habitat for several marine predators (southern elephant seals: Labrousse et al., 2015, crabeater seals: Burns et al., 2004, emperor penguins [*Aptenodytes forsteri*]: Rodary, Bonneau, Le Maho, & Bost, 2000, Weddell seals [*Leptonychotes weddellii*]: Heerah et al., 2013). Sea‐ice serves as a substrate for sea‐ice algae and a refuge from other predators, but also represents a physical barrier that may constrain the movements of air‐breathing animals and their access to favorable foraging grounds (Hindell et al.*,* 2016; Tynan et al., 2009). Moreover, the interplay between bathymetric features and other physical components such as the hydrological circulation is likely to influence prey distribution and availability (Chapman, Ribic, & Fraser, 2004; Heerah et al., 2013; Nicol, Meiners, & Raymond, 2010).

East Antarctica is defined as the region of the southern Indian and Pacific Ocean sectors between 80 and 160°E (Nicol et al., 2000, 2010). Across this region, physical and biological features vary considerably in space and time, largely influenced and delimited by the southern boundary of the Antarctic Circumpolar Current (Nicol et al., 2010; Tynan, 1998). These spatiotemporal habitats have different prey assemblages, distribution, and availability, which ultimately affect the biology and foraging behavior of focal predators.

Emperor penguins and Weddell seals are the only air‐breathing, warm‐blooded predators remaining in the high‐latitude (South of 60°S) Antarctic fast‐ice year‐round (Burns & Kooyman, 2001). The fact that emperor penguins brood their egg and chick while fasting throughout the Antarctic winter is the best illustration of their adaptation to this extreme environment. The Weddell seal, on the other hand, is the only deep‐diving—up to 900 m deep (Heerah et al., 2013), Antarctic air‐breathing marine predator adapted to breathe through holes in year‐round ice cover (Kooyman, 1981; Stirling, 1969). These adaptations enable Weddell seals to access the under‐ice habitat and to forage on a range of prey such as fish, cephalopods, and crustaceans unavailable to other air‐breathing predators at this time (Ainley & Siniff, 2009; Burns, Trumble, Castellini, & Testa, 1998; Kooyman, 1981; Lake, Burton, & van den Hoff, 2003).

Studying Weddell seal behavior and ecology during their postmolt, overwinter foraging trip (February–October referred to as winter in the present study) is especially insightful for understanding how the biotic environment and abiotic environment influence the individual and population characteristics observed in spring and summer (Chambert, Rotella, & Garrott, 2015; Testa, 1994). This is because the resources accumulated overwinter directly affect the vital rates (i.e., survival and fecundity). Only one study, to our knowledge, has quantified Weddell seal postmolt foraging behavior in response to the Antarctic overwinter environmental conditions (Heerah et al., 2013), and none have compared regional behavior differences. Taking a comparative approach highlights key foraging strategies that Weddell seals have evolved/learned to maximize food acquisition, which would not be apparent from studying a single study site, and allows a broader understanding of the foraging strategies of sea‐ice‐obligate species. Our study compares the overwinter postmolt foraging behavior of Weddell seal populations from two widely separated regions and aims to answer two main questions: (1) “What are the foraging strategies adopted by Weddell seals in contrasting environments?” and (2) “Which environmental parameters influence their behavior?”

## 2 Materials and Methods

### 2.1 Instrumentation

We studied Weddell seal behavior at two sites in East Antarctica: Dumont D'Urville (DDU; 66°40′S 140°E) and Davis (68°58′S 77°97′E) during their overwinter foraging trips (DDU: 2007–09, Davis: 2006–07 and 2011). Adult Weddell seals were captured after their annual molt in February at DDU (*N*_female_ = 9 and *N*_male_ = 3, length: 230 ± 3 cm and mass: 284 ± 17 kg) and at Davis (*N*_female_ = 18, *N*_male_ = 2, length: 240 ± 3 cm and mass: 365 ± 13). Similar capture and tagging procedures were used at both sites and are fully described in Heerah et al. (2013). Satellite‐relayed data loggers (SRDLs) were head‐mounted on the Weddell seals, recording their movements and diving behavior throughout the whole winter. Only seals for which the tag transmitted for longer than 90 days were included.

### 2.2 Argos locations filtering, track simulations, and environmental variable extraction

We filtered the ARGOS locations using (1) a swim speed filter with the maximum speed set to 20 km/hr, which resulted in the removal of 15% of the ARGOS locations, and (2) a Kalman filter, which accounted for location error according to their assigned ARGOS location class (R package “crawl”; Johnson, London, Lea, & Durban, 2008). The resulting correlated random walk models (CRWM) were then used to predict a location (and estimated uncertainty) for each dive (Johnson et al., 2008). To account for location error when extracting environmental variables, we used the fitted CRWM to create a dataset of 100 simulations of each dive location and individual seal (Johnson et al., 2008). The 100 values for the bathymetry, slope (see Appendix S1), and sea‐ice concentration associated with each possible dive location were first extracted and then averaged, giving a mean value and its standard deviation for each location along the mean track. We also calculated the distance between each dive and the nearest coastline. Sea‐ice concentration values were used to calculate “the distance to ice edge” and “sdice25,” which is an index of the spatial variation of sea‐ice concentration in the vicinity of each dive. Details of the environmental datasets used and calculation of the slope, “distance to ice edge,” and “sdice25” variables are provided in Appendix S1. The influence of time of day, and therefore light intensity, on Weddell seals’ diving behavior was accounted for after Heerah et al. (2013).

### 2.3 Diving behavior

#### 2.3.1 Data collected from the tags

The SRDLs recorded and transmitted (Fedak, Lovell, McConnell, & Hunter, 2002) a total of 142,294 dive profiles for the 32 focal Weddell seals (4,447 ± 257, mean ± *SE*). However, 57,347 dives (~40% of the dataset) were excluded from our analyses. First, they corresponded to dives within 20 m of the surface (27% of the dives representing only 5.4% of total time spent diving) we chose to not consider (see also Heerah et al., 2013). The other excluded dives (13% of the dives) resulted from rounding errors (e.g., equal successive times), dive cut issues, and unrealistically deep dives (>1,500 m).

For the remaining 84,947 dives, we calculated the difference between the maximum dive depth and corresponding bathymetry (hereafter “depth difference”). Twenty‐six percentage of dive depths were deeper than the bathymetry, likely due to the combined error of both bathymetry and seal positions. The depth difference was normally distributed with a mode between −30 and 30 m for dives from DDU and a mode between −50 and 50 m for dives from Davis, suggesting that these modes (hereafter “error threshold”) represent dives to the seafloor (i.e., benthic dives) (Heerah et al., 2013; Labrousse et al., 2015). Dives 30 and 50 m deeper than the bathymetry at DDU and Davis, respectively, were removed from the dataset (DDU: 4% of the dives, Davis: 7% of the dives). Dives apparently on land, likely due to dive position error, were also removed from the dataset (4% of the dives at Davis and DDU). Dives were separated into two types: (1) benthic dives (i.e., maximum dive depths within the error threshold, dives to the seafloor) and (2) pelagic dives (i.e., maximum dive depths shallower than the error threshold, dives in the water column).

#### 2.3.2 Calculation of a vertical foraging metric: hunting time

Following Heerah, Hindell, Guinet, and Charrassin (2015), we calculated the vertical rate of change (i.e., vertical velocity, m/s) for each of the five dive segments that made up a dive. Segments with vertical velocities ≤0.4 m/s were defined as “hunting” segments, whereas segments with vertical velocities >0.4 m/s were defined as “transit” segments (Heerah et al., 2015). The total time spent in the “hunting” segments within a dive was used as a vertical foraging effort metric (which includes the time spent searching, pursuing, and potentially catching a prey as discussed in Heerah, Hindell, Guinet, & Charrassin, 2014; Heerah et al., 2015).

### 2.4 Movement pattern analyses: integration of a vertical foraging metric

To identify behavioral changes along a seal's horizontal path, we used a track‐based method adapted from first‐passage time (FPT) analysis (Fauchald & Tveraa, 2003). Our method is similar to FPT analyses, except that instead of measuring the time required to cross a circle of given radius, we summed the total time spent hunting within that circle. This adaptation allowed us to identify behavioral changes at depth (at the optimal spatial scale for each individual) and is termed the first hunting time (FHT; see details of the analysis in Appendix S2, Figs S1 and S2).

Fauchald and Tveraa (2003) defined search areas as the areas associated with the longest FPT. Like Thums, Bradshaw, and Hindell (2011), we used the distribution of FHT density estimates to find a time threshold discriminating the mode of lower FHT values (i.e., “transit”) from all other higher modes (i.e., “hunting”) (Appendix S2 and Fig. S1). Dives with FHT values below the time threshold were defined as “transit” dives, whereas dives with FHT values above the time threshold were considered “hunting” dives. We then plotted daily FHT at the optimal spatial scale for each individual and dives associated with intensified hunting at depth (Fig. S1d, e).

### 2.5 Statistical analysis

We fit a series of generalized mixed‐effect models with multivariate normal random effects, using penalized quasi‐likelihood (R package “MASS,” function “glmmPQL,” Venables & Ripley, 2002) to examine the relationship between our binary behavioral response variable (“transit” vs. “hunting” dives) and the explanatory variables (i.e., temporal [day of year], site, and environmental factors [bathymetry, slope, sea‐ice concentrations, distance to ice edge, *sdice25*]). Individual seal was included as a random term on the intercept. We started with a full model that included all environmental variables and meaningful variable interactions (i.e., influence of site). We then implemented a stepwise procedure to remove nonsignificant variables with the threshold set at *p*‐value < .05 (Zuur, Ieno, Walker, Saveliev, & Smith, 2009; see Appendix S3 for details on model selection, procedure, and validation).

Wilcoxon rank‐sum tests were used to compare average movement, behavioral, and environmental metrics between (1) sites, (2) behavioral modes (i.e., hunting and transit) within each site and (3) each behavioral mode between sites. Samples consisted of the means of the metric of interest for each individual separately and were therefore independent.

## 3 Results

### 3.1 Tag performance

Female Weddell seals from DDU (2007: *n* = 13, 2008: *n* = 3, 2009: *n* = 6) and Davis (2006: *n* = 2, 2007: *n* = 3, 2011: *n* = 15) were tracked for 183 ± 13 days (mean ± *SE*, max: 242 days, 2007: 211 ± 17, 2008: 217 ± 18, 2009: 145 ± 13) and 158 ± 7 days (max: 199 days, 2006: 136 ± 42, 2007: 161 ± 22, 2011: 161 ± 8), respectively, from late February to mid‐October. Most data were collected between March and August. A total of 31,402 dives (16 ± 0.9 dives per day, max: 22) were used for analysis for seals from DDU and 51,901 dives (19 ± 0.9 dives per day, max: 27) for seals from Davis.

### 3.2 Identification of horizontal area‐restricted search

**Figure 1:**
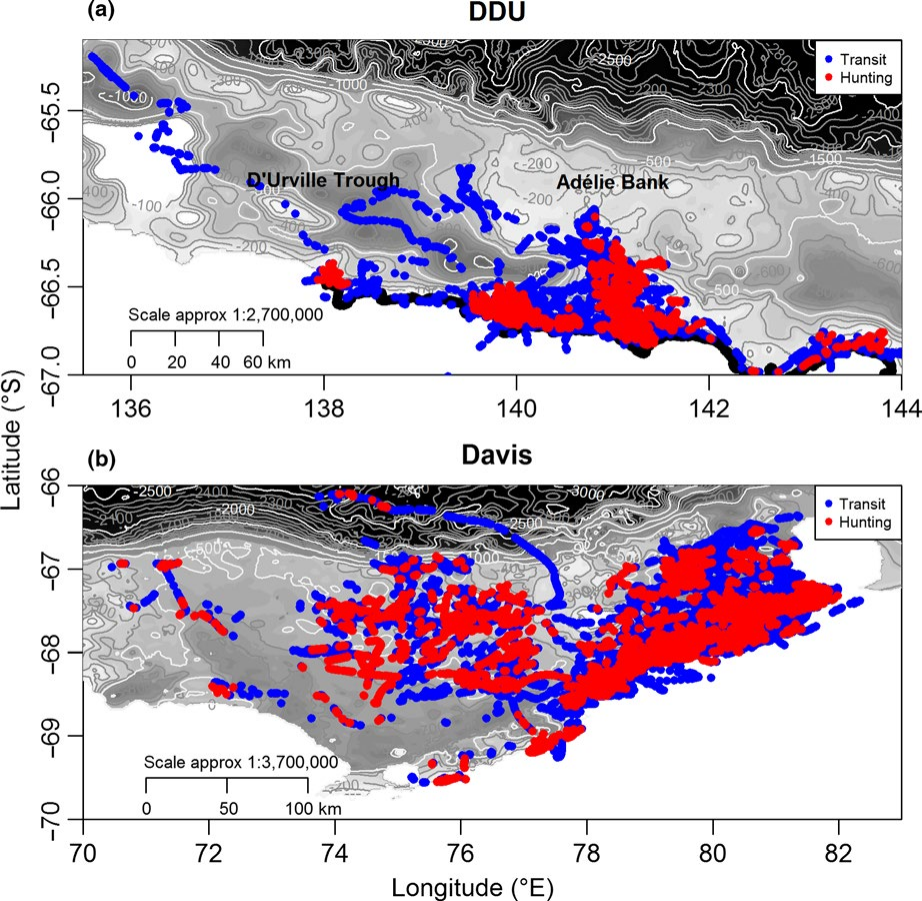
Dives of seals from each colony assigned with a behavioral mode according to first hunting time analysis (i.e., transit and hunting): (a) Dumont D'Urville and (b) Davis colonies over multiple years

All seals exhibited horizontal ARS behaviors, although the optimal spatial scales of their search patterns varied among individuals. Optimal spatial scales derived from FHT analysis ranged from 0.5 to 15 km and were on average similar at DDU (5.2 ± 1 km) and Davis (6.4 ± 1.2 km) (unilateral Wilcoxon test, *p*‐value > .05). However, seals from DDU spent two times longer (*w* = 182, *p*‐value < .05) hunting at their optimum scale (25 ± 6 hr, max: 73 hr) compared to seals from Davis (12 ± 4 hr, max: 76 hr). Similarly, the FHT threshold, used to discriminate “transit” from “hunting” behavior, of seals from DDU was almost twice as long (*w* = 183, *p*‐value < .05; 13 ± 4 hr, max: 56 hr) as for seals from Davis (7 ± 2, max: 46 hr) despite the proportions of transit and hunting dives being similar at both sites (*p*‐value > .05). Seals from DDU performed 45 ± 4% (max: 73%) transit dives and 55% ± 4% (max: 71%) hunting dives (Figure 1a), while seals from Davis performed 49% ± 2% (max: 71%) transit dives and 48% ± 3% (max: 78%) hunting dives (Figure 1b).

### 3.3 Horizontal movement patterns

Seals from both sites remained on the Antarctic continental shelf throughout the winter (Figures 1 and S3). However, there were clear differences in the scales of movement among individuals within each site and between the two sites (Fig. S3). For seals from DDU, the mean distance from the shore and deployment site for each seal ranged from 1 ± 0.03 to 25 ± 0.3 km (mean: 8 ± 2 km, max: 78 km) and from 2 ± 0.05 to 74 ± 1 km (mean: 30 ± 6 km, max: 259 km), respectively (Figures 1a and S3a). On average, these seals traveled 4 ± 1 km/day (max: 75 km/day), although average distances for each seal ranged from 0.5 ± 0.04 to 12 ± 1 km/day. Most seals remained coastal, in the vicinity of the site where the seals were originally captured; however, three individuals traveled beyond this zone: one to the D'Urville Trough (wd3‐CTD3‐07), one to the western (ct47‐B‐09), and one to the eastern (ct47‐D‐09) parts of the shelf in the study area (Fig. S3a).

In contrast, seals from Davis traveled three times further their DDU compatriots (*p*‐value < .05; Figure 1b). Average travel distances ranged from 4 ± 0.05 km to 116 ± 1 km (mean: 30 ± 0.6 km, max: 293 km) from the coast and 18 ± 0.1 to 169 ± 1 km (mean: 88 ± 11 km, max: 372 km) from the deployment site. Overall, seals traveled 11 ± 1 km/day (max: 144 km/day), although there was considerable variation between individual seals and average distances ranged from 3 ± 0.3 to 21 ± 2 km/day. Most seals from Davis traveled to the northeastern part of the shelf although five traveled west to the middle shelf area and one traveled north (wd4‐880‐11), diving over the shelf break and in areas deeper than 2,000 m (Fig. S3b).

At DDU, hunting was concentrated in four regions, while transit behavior occurred across a much broader spatial domain (Figure 1a). In contrast, at Davis hunting dives were diffuse across a wide area, and rather than being spatially distinct, the two dive types overlapped (Figure 1b).

### 3.4 Diving behavior

#### 3.4.1 Dive metrics

On average, seals from DDU made shallower dives (mean: 113 ± 0.6 m, max: 904) than seals at Davis (mean: 174 ± 0.6 m; max: 1,094 m) (unilateral Wilcoxon test: *w* = 193, *p*‐value < .05). Similarly, mean dive depths were different between sites during transit (DDU: 95 ± 0.7 m, max: 166 m; Davis: 156 ± 0.9 m, max: 242 m) and hunting (DDU: 91 ± 0.5 m, max: 154 m; Davis: 144 ± 0.8 m, max: 241 m; *p*‐value < .01).

Mean dive durations were on average shorter at DDU than at Davis (*w* = 169, *p*‐value < .05) and lasted 11.5 ± 0.03 min (max: 84 min) and 12.5 ± 0.03 min (max: 65 min), respectively. A similar trend was observed for transit (DDU: 10.1 ± 0.05 min, max: 40 min; Davis: 11.6 ± 0.04 min, max: 55 min) and hunting dives (DDU: 11.2 ± 0.05 min, max: 84 min; Davis: 12.2 ± 0.04 min, max: 62 min; *p*‐value < .01). At both sites, dive depths and durations did not differ while comparing transit and hunting modes (*p*‐value > .05).

The time spent hunting within each dive was similar between the two sites (*p*‐value > .05). Seals spent 8 ± 0.02 min (max: 84 min) and 7.5 ± 0.02 (max: 62 min) hunting within a dive representing 55% ± 4% (max: 71%) and 48% ± 3% (max: 78%) of the total time spent diving at DDU and Davis, respectively. At both locations, hunting time within each dive was 1.2 times longer (*p*‐value < .05) in hunting dives (DDU: 8.7 ± 0.04 min; Davis: 8.1 ± 0.04 min) than in transit dives (DDU: 7.4 ± 0.05 min; Davis: 6.9 ± 0.03 min).

#### 3.4.2 Pelagic versus benthic dives

At both sites, seals made predominantly pelagic dives: 67% ± 6% (max: 92%) and 72% ± 3% (max: 89%) at DDU and Davis, respectively, than benthic dives. However, despite the general predominance of pelagic dives, two individuals, one from DDU and the other one from Davis, made more benthic dives (75% and 69% at DDU and Davis, respectively) than pelagic dives.

**Figure 2:**
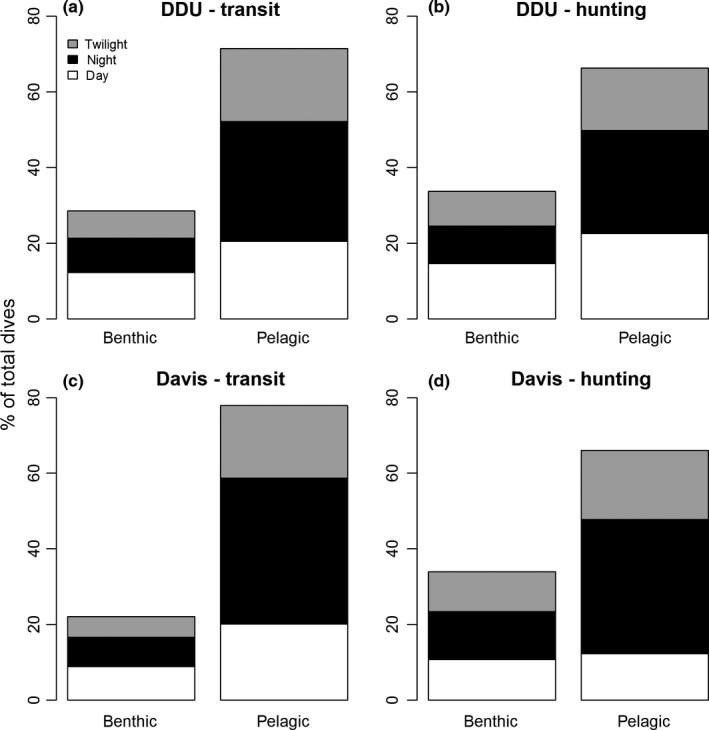
Proportion of benthic and pelagic dives performed by seals from Dumont d'Urville (DDU; a and b) and Davis (c and d) according to behavioral mode (i.e., transit or hunting) and the time of the day (day, twilight, and night). Data were pooled from multiple years and seals for each colony. (a) 71% of total transit dives at DDU were pelagic (day: 20%, night: 32%, twilight: 19%), whereas 29% were benthic (day: 12%, night: 9%, twilight: 8%). (b) 66% of total hunting dives at DDU were pelagic (day: 23%, night: 27%, twilight: 16%), whereas 34% were benthic (day: 15%, night: 10%, twilight: 9%). (c) 78% of total transit dives at Davis were pelagic (day: 20%, night: 39%, twilight: 19%), whereas 22% were benthic (day: 9%, night: 8%, twilight: 5%). (d): 66% of total hunting dives at Davis were pelagic (day: 12%, night: 36%, twilight: 18%), whereas 34% were benthic (day: 11%, night: 13%, twilight: 10%)

Despite the similarities in the general dive types at DDU and Davis, there were regional differences between dive types in relation to behavioral mode. At DDU, dives were pelagic regardless of whether the seals were in transit (71%) or hunting mode (66%) (Figure 2a, b). For both behavioral modes, pelagic dives mostly occurred at night, whereas benthic dives mainly occurred during the day (Figure 2a, b). In contrast, at Davis 78% of transit dives were pelagic and 22% were benthic (Figure 1c, d), while for hunting dives 66% were pelagic and 34% benthic (Figure 1c, d). For both behavioral modes, pelagic dives mostly occurred at night, whereas benthic dives occurred almost equally during day, twilight, and at night (Figure 2a, b).

### 3.5 Influence of the environment on behavior and habitat use

**Figure 3:**
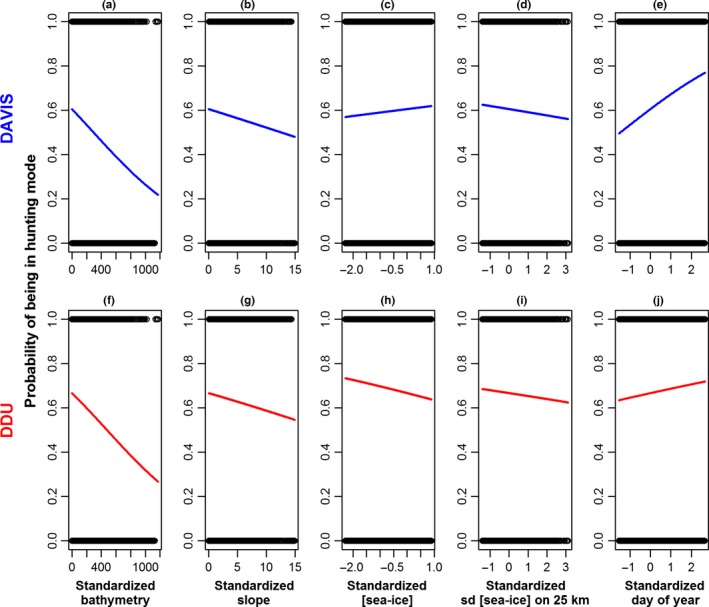
The relationship between hunting mode likelihood and bathymetry (a and f), seafloor slope (b and g), sea‐ice concentration (c and h), sea‐ice spatial variability over 25 km around each seal's location (d and i) and day of year (DOY, e and j) from our generalized mixed‐effect model (GLMM). Relationships are shown for Davis (a–e) and DDU (Dumont D'Urville, f–j). Explanatory variables were standardized to allow comparison of their slope coefficients. Confidence intervals were plotted, but are too narrow to be visible on the graph

At DDU and Davis, the likelihood of seals being in hunting mode was related to, in order of importance, the bathymetry, the day of year, the slope as well as sea‐ice concentration and spatial variability over 25 km (Table 1, Figure 3). Conversely, the distance to open water areas did not appear to influence behavioral mode. Overall, these relationships were consistent among individuals given that the intercept of the random effect was near zero (Table 1).

**Table 1:** Generalized mixed‐effect model outputs

**Model: ARS ~ bathymetry + slope + ice_conc + sdice25 + DOY + factor(colony) + factor(colony) × DOY + factor (colony) × ice_conc**					
*n* observations: 20,015					
*n* individuals: 32					
**Random effects: ~1|seal ID**					
	Intercept	Residual			
Std dev:	0.000124294	0.9877119			
	**Value**	**Std. error**	***df***	***t*** **‐value**	***p*** **‐Value**
Intercept	0.4224096	.07019963	19,976	6.017262	.00000
Bathymetry	−0.0014502	.00013745	19,976	−10.550321	.00000
Slope	−0.0339922	.00970807	19,976	−3.501437	.00050
Ice_conc	0.0644655	.03143822	19,976	2.050545	.04030
Sdice25	−0.0592402	.02593525	19,976	−2.284157	.02240
DOY	0.2915468	.05356549	19,976	5.442811	.00000
Factor(colony)DDU	0.2673167	.10744383	30	2.487966	.01860
DOY:factor(colony)DDU	−0.199608	.09011438	19,976	−2.215052	.02680
Ice_conc:factor(colony)DDU	−0.2048494	.05532356	19,976	−3.702751	.00020

Generalized mixed‐effect model output for the final model (on dives from both colonies) including each of the significant fixed explanatory variables (bathymetry, bathymetric slope, sea‐ice concentration [ice_conc], sea‐ice variation within a 25‐km radius of each dive [sdice25], day of year [DOY]). ARS is the binomial response variable: “transit” or “hunting.” The colony (Davis vs. Dumont D'Urville [DDU]) was used as a factor, and its interaction with the sea‐ice concentration and the day of year was significant. Individuals were used as random effect on the intercept.

**Figure 4:**
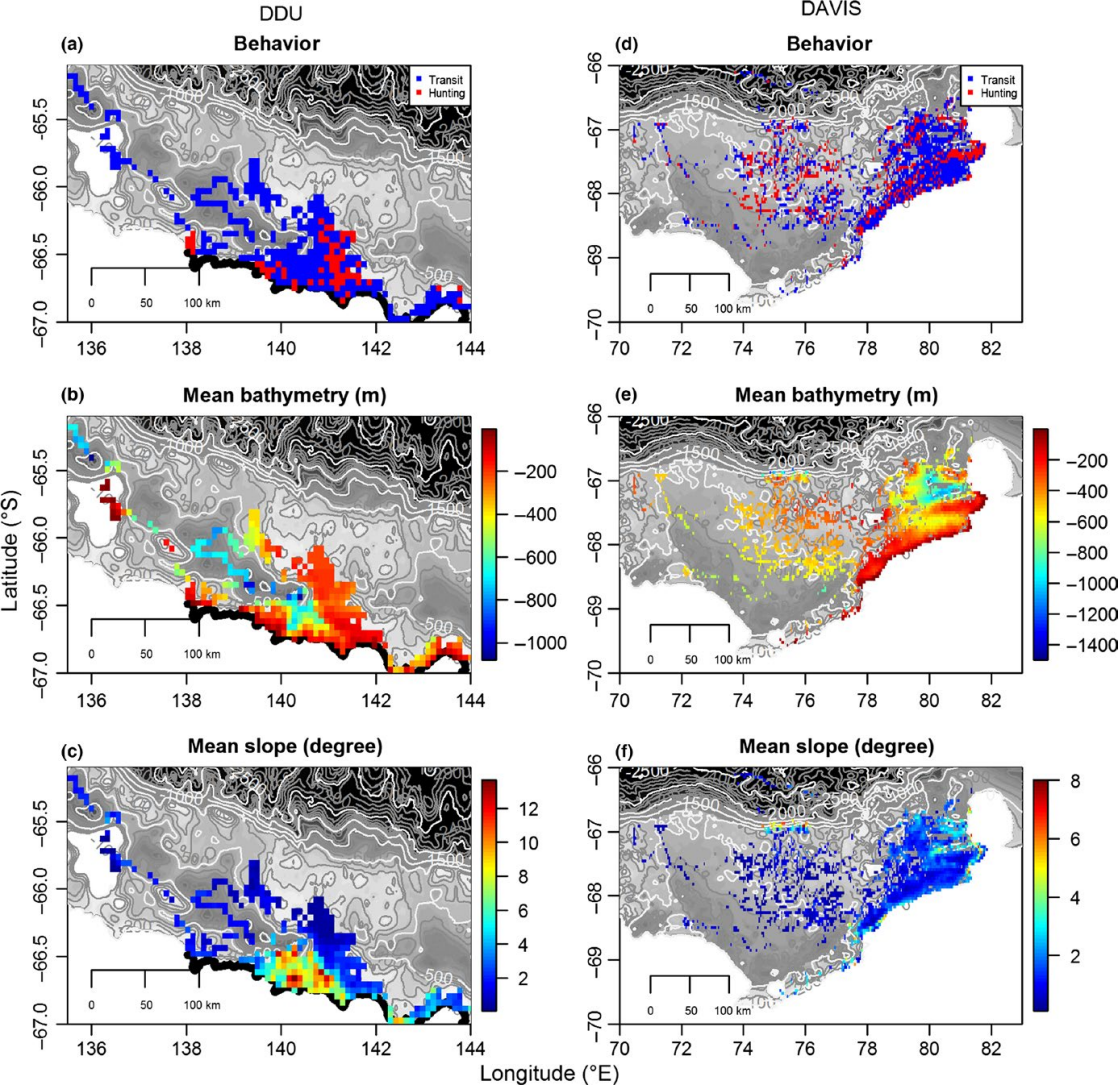
Maps of gridded dive locations (5 × 5 km) for seals from Dumont D'Urville (DDU; a–c) and Davis colonies (d–f). Values within each cell are expressed as the most frequent behavioral mode (a and d), and average value of topographic features according to bathymetry (b and e) or bathymetric slope (c and f) within the 25 km² of each gridded location

**Figure 5:**
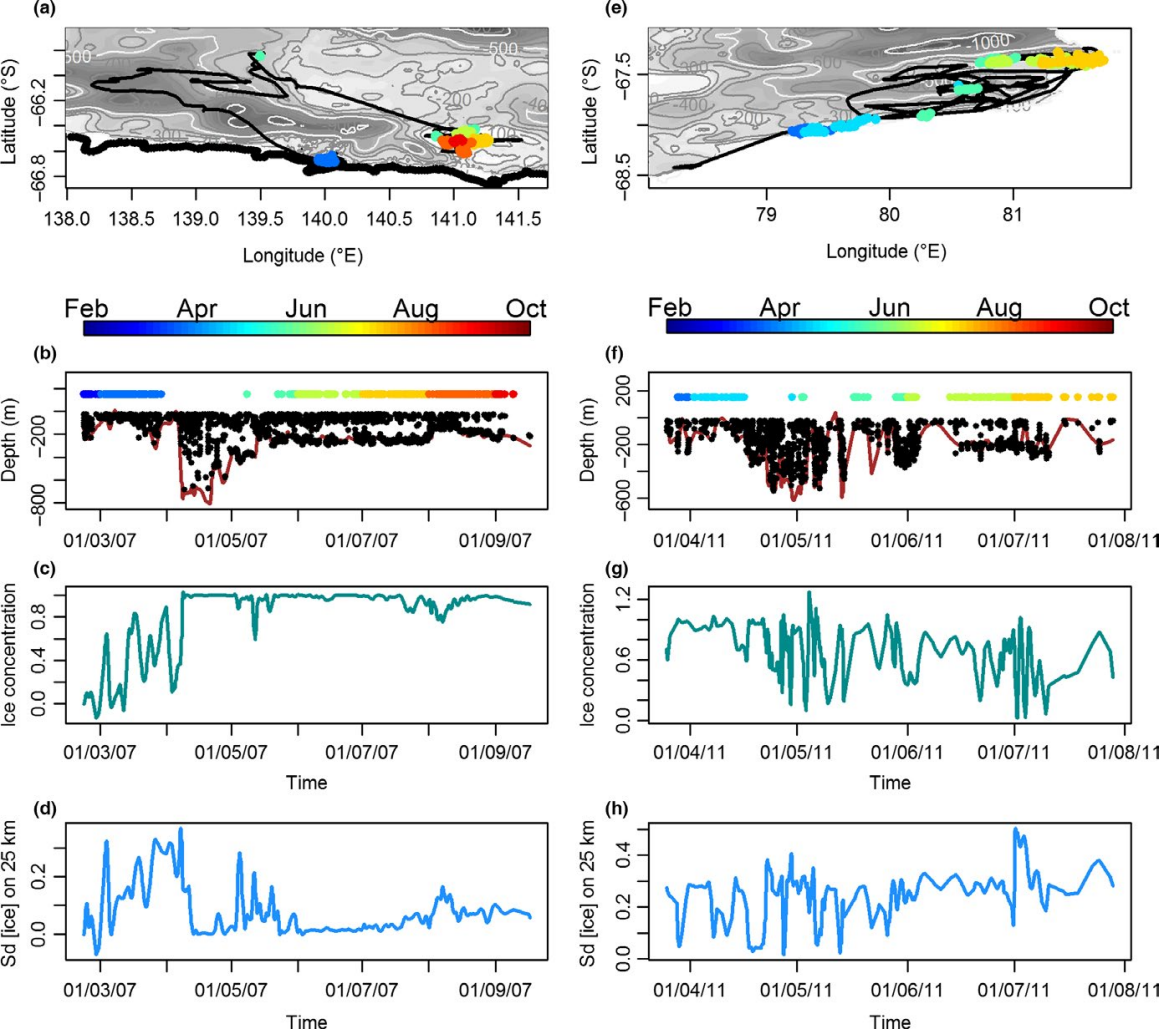
Temporal variations of movement patterns and habitat use of an individual seal from each colony: (a–d) Dumont d'Urville and (e–h) Davis. Hunting dive locations are color‐coded according to the time of the year (a, b, e, f). Bathymetry, sea‐ice concentration, and its variation within a 25‐km radius of each dive (*SD* [ice] on 25 km) were extracted and calculated for each dive (see Section 2.3)

At both sites, the probability of being in hunting mode increased with day of year and was stronger at Davis (coef = .29, *p*‐value < .01) than at DDU (coef = .09, *p*‐value < .05) (Figures 3 and 5). Conversely, at both sites, the probability of being in hunting mode was negatively related to the bathymetry (coef = −.001, *p*‐value < .001) and the seafloor slope (coef = −.03, *p*‐value < .001). The seals appeared to concentrate most of their foraging effort in shallow waters on the edge of deeper canyons and depressions (Figures 4 and 5a, b, e, f). Seals from DDU used waters that were 174 ± 1 m deep associated with a seafloor slope of 5 ± 0.02 degrees when hunting, whereas they used areas of 226 ± 2 m (max: 1,184) and 6 ± 0.03° (max: 18°) while in transit (Figure 4a–c). At Davis, areas used were overall deeper than in DDU and were of 310 ± 2 and 386 ± 2 m associated with seafloor slope of 1 ± 0.01° and 1 ± 0.01°, in hunting and transit modes, respectively (Figure 4d, f).

The influence of sea‐ice concentration on behavioral mode was weak and differed between the study sites. The probability of being in hunting mode was positively related to sea‐ice concentration at Davis (coef = .06, *p*‐value < .05), but negatively related to it at DDU (coef = −.14, *p*‐value < .01; Table 1, Figure 3). However, at both sites, the probability of being in hunting mode decreased with increasing sea‐ice spatial variability (over 25 km; coef = −.06, *p*‐value < .05; Table 1, Figure 3). Seals from Davis used sea‐ice concentrations of 77% ± 2% (median: 84%) and 74% ± 30% (median: 84%) that varied over 25 km of 50% ± 10% and 51% ± 10% when in hunting and transit modes, respectively. Sea‐ice concentrations at DDU were 69% ± 0.1% (median: 87%) and 73% ± 0.1% (median: 91%) associated with variations (over 25 km) of 44% ± 0.1% and 45% ± 0.1% in hunting and transit modes, respectively. Overall, at DDU, the sea‐ice concentrations and spatial variations did not vary much over winter (Figure 5c, d) and are indicative of a fast‐ice coastal area, whereas at Davis highly variable sea‐ice patterns over winter (Figure 5g, h) reveal typical coastal polynya characteristics (M. Vancopenolle, pers. Com., V. Andrews‐Goff *unpublished*). These observations are supported by the pluri‐annual occurrence of a coastal polynya (68.7°S 81.6°E) at Davis, which was also the main hunting area of 12 individuals (Fig. S4).

## 4 Discussion

Weddell seals are unique among Southern Ocean phocids in that they spend all winter in the high Antarctic (South of 60°S). During winter, the sea‐ice growing forms a major obstacle to the seals that need to accumulate resources during the Austral winter to support breeding in the following spring. How the seals balance the need to breathe and dive for food in areas of dense ice remains a vexing question, despite many years of study across several Antarctic locations (such as the Ross Sea: Burns, Castellini, & Testa, 1999; Burns & Kooyman, 2001; Kooyman, 1981; Testa, 1994 and Prydz Bay: Andrews‐Goff, Hindell, Field, Wheatley, & Charrassin, 2010; Lake, Burton, & Wotherspoon, 2006; Lake, Wotherspoon, & Burton, 2005). To quantify how seals acquire the resources needed to support their capital breeding strategy, we quantified the diving behavior of two populations of Weddell seals at sites with contrasting physical attributes.

### 4.1 Methodological discussion

State‐space models (SSMs) are a powerful tool used to detect ARS (ARS) patterns in a range of species (Dragon, Bar‐Hen, Monestiez, & Guinet, 2012; Jonsen, Myers, & James, 2007). However, Weddell seals’ small movement scales, combined with lengthy periods of inactivity, make it difficult for SSMs to distinguish behavioral modes (Andrews‐Goff, 2010). Given this, FPT may provide a more robust quantification of foraging behavior (Fauchald & Tveraa, 2003). However, both FPT and SSM analyses rely on the 2D track of the animals and cannot discriminate between foraging and other activities, such as haul‐out periods between dives. To overcome these limitations, we incorporated a “hunting” metric (Heerah et al., 2015). Being able to track and quantify the spatiotemporal abundance of prey from a simple metric like FHT has broad implications for using predator behaviors to study the biology of the hard to study prey (e.g., meso‐pelagic fish), of predators like seals, when direct observations are not possible (Davis, Fuiman, Williams, Horning, & Hagey, 2003; Davis et al., 1999; Fuiman, Davis, & Williams, 2002). Importantly, it also allows us to compare robustly diving and foraging behavior between sites.

### 4.2 Dense coastal ice: a primary habitat for Weddell seals

Seals from both DDU and Davis remained in coastal highly concentrated ice areas over winter. However, it is clear that Weddell seals exploited their sea‐ice environment differently in response to local sea‐ice conditions. For instance, sea‐ice conditions at DDU were less variable spatially and temporally than at Davis, and accordingly, the seals ranged less than seals at the Davis site (Massom et al., 2013). Moreover, Weddell seals from DDU tended to spend more hunting time in areas with less concentrated ice, while the opposite was observed for seals from Davis. At both locations, seals displayed more hunting time in regions with less variable sea‐ice (within 25 km). While a small coastal polynya at the Davis site seemed to attract several foraging individuals, most hunting dives (as well as transit dives) were still associated with highly concentrated sea‐ice at both sites. In addition, the foraging behavior at both sites was not influenced by distance to open water areas.

Polynyas were not an important sea‐ice feature for the seals. Rather, Weddell seals are more likely to be influenced by sea‐ice thickness and stability (rather than just sea‐ice concentration). The ice needs to be thick enough to ensure a stable haul‐out platform, but thin enough to allow the seals to maintain their breathing holes without compromising survival through damage to their teeth (Lake et al., 2005; Stirling, 1969). Also, it may be that the seals are responding to smaller features within the fast‐ice environment such as the presence of perennial tide cracks that are invisible at the coarse resolution of the sea‐ice data available to us. Indeed, tide cracks are an important determinant of survival and reproductive output in Weddell seals (Chambert, Rotella, & Garrott, 2012); when cracks are absent, survival and reproductive output decreases. In response to this high selection pressure, Weddell seals are likely to remember the locality of tide cracks and rely on them from year to year (Kooyman, 1981). This is supported by the pluri‐annual site fidelity observed in our studies and their close proximity to land where tidal action favors tide cracks formation (Lake et al., 2005). This pattern was even more pronounced in DDU where sea‐ice conditions are less variable both in space and in time, resulting in fewer suitable sites for both breathing and foraging compared to those at Davis.

### 4.3 Foraging strategies of Weddell seals

Despite sea‐ice representing an obstacle for accessing the water, the neritic ice‐covered area also represents a reliable source of food during winter if it can be accessed (Tynan et al., 2009). It is likely that Weddell seals have evolved or learned behavioral tactics in order to meet their food requirements within the range of constraints imposed by the environment and their physiological abilities (e.g., finding a breathing hole [see above], minimizing travel costs, targeting prey within their depth range).

#### 4.3.1 Foraging from a breathing hole

At both study sites, the optimal hunting scale (~5–6 km) corresponded to the range of distances that a seal could travel underwater between breathing holes in a single breathe (Kooyman, 1981). Several authors proposed that Weddell seals foraging beneath fast‐ice will dive from a breathing hole until resources within its accessible radius become depleted (Hindell, Harcourt, Waas, & Thompson, 2002; Kooyman, 1981; Testa, Siniff, Ross, & Winter, 1985); so it is likely that seals travel between a network of holes close to each other. Although the optimal hunting scales were similar at Davis and DDU, seals from Davis spent half the time hunting in an area of a given radius. This could be due to faster prey depletion (because there are fewer prey or higher intraspecific competition) in a given area in Davis or it could be related to different environmental conditions that influence prey availability and/or accessibility. Based on our observations, the latter is more plausible as we showed contrasting sea‐ice conditions between DDU and Davis, in which Davis sea‐ice conditions were more variable. Traveling between holes represents a risk of disorientation and/or reaching an area covered of thick ice that would be costly to open and maintain. To maximize resource intake within a patch, a predator's residence time is related to the cost of travel to the patch and the quality of that patch (e.g., abundance and/or prey type). For Weddell seals, the risk of traveling to another breathing hole is as an additional cost to the total (horizontal + vertical) travel cost of reaching a patch of prey. In an environment where travel costs between prey patches are higher (e.g., DDU where sea‐ice is dense and less variable), we could expect a predator to increase its time spent searching for prey in a given patch; accordingly, the seals at DDU spent twice much time hunting in a given area than in Davis where the sea‐ice is more variable.

#### 4.3.2 Inference on Weddell seals’ diet from diving behavior

The preferred foraging depths of deep‐diving predators are influenced by their diving capacity and prey distribution (Burns & Kooyman, 2001; Davis et al., 1999; Watanabe, Mitani, Sato, Cameron, & Naito, 2003). Weddell seals at both sites used the entire water column, performing benthic and pelagic dives. Despite the similarities in general diving behavior between sites, there was substantial inter‐ and intrasite variability. The complexity and individual variability of the seal's diving behavior most likely reflects Weddell seals’ opportunistic foraging behavior and cosmopolitan diets (Davis, Fuiman, Madden, & Williams, 2013; Davis et al., 2003; Heerah et al., 2014, 2015). Weddell seals feed on a variety of pelagic (e.g., *Pleurogramma antarcticum*,* Dissostichus mawsoni* and squids) and benthic prey (e.g., *Trematomus* spp. and crustaceans; Ainley & Siniff, 2009; Burns et al., 1998; Goetz, Burns, Hückstädt, Shero, & Costa, 2016).

Pelagic dives occurred mostly at night, whereas benthic dives were most prevalent during the day, suggesting that Weddell seals follow the diel migration of their pelagic prey (such as *P. antarcticum*, Fuiman et al., 2002). This temporal segregation was most pronounced at DDU. At Davis, while pelagic dives mainly occurred at night, benthic dives occurred equally during the day and at night, which suggests that seals at Davis have a more varied diet than the seals at DDU. This is likely due to the larger range used by the seals from Davis. For instance, previous studies reported Davis Weddell seals foraging in the southern fjords and inshore areas mostly consumed benthic fishes and prawns, whereas in the northern and offshore areas, their diet was dominated by *P. antarcticum* (Lake et al., 2003). In variable and unpredictable environments, animals should display generalist behaviors and have cosmopolitan diets (Laidre et al., 2008). The variable foraging behaviors we observed could be a strategy evolved to increase survival through and reproductive success after Antarctic winter (i.e., heavy sea‐ice, darkness, and associated decrease in productivity). For instance, if a target prey species is depleted in the vicinity of a breathing hole, but the seal is unable to move to another hole due to heavy sea‐ice conditions, it may likely switch to other prey species.

#### 4.3.3 Importance of the seasonal advance on foraging

The probability of being in hunting mode increased with seasonal advance during winter, which coincides with gestation and the need to build up lipid stores for the breeding season (Kooyman, 1981; Wheatley, Bradshaw, Harcourt, & Hindell, 2008). To maximize their energy intake, Weddell seals can (1) minimize the costs associated with travel between prey patches as sea‐ice thickens during winter by increasing their hunting effort in a given area and (2) by favoring environmental conditions likely to be associated with increased prey availability and accessibility.

#### 4.3.4 Environmental parameters influencing the behavior of Weddell seals

In our study, Weddell seals from both sites were more likely to show hunting behavior in relatively shallower areas where the bathymetry interacts with other physical features such as the water masses, and ultimately the sea‐ice. The troughs and depressions surrounding the foraging grounds of the seals could facilitate the upwelling of the warmer, macronutrient‐enriched modified Circumpolar Deep Water (mCDW) onto shallower areas (Prézelin, Hofmann, Mengelt, & Klinck, 2000; Tynan, 1998). The importance of this water mass to the Antarctic ecosystem has been highlighted in previous studies (Hindell et al., 2016) and is known to be associated with the foraging behavior of other top predators such as Southern elephant seals while foraging on the peri‐Antarctic shelf break (Labrousse et al., 2015). This nutrient‐enriched water mass stimulates productivity (Prézelin et al., 2000), thereby attracting zooplankton and fish providing a predictable source of food for top predators. It is not known whether this holds true for winter because of limited light availability; however, juvenile *P*. *antarcticum* are found in association with this water mass on the continental shelf (La Mesa et al., 2010). Interactions between bathymetry and water mass boundaries may also aggregate seal prey (Ribic, Chapman, Fraser, Lawson, & Wiebe, 2008; Zhou & Dorland, 2004). Finally, the warmer mCDW could interact with sea‐ice and facilitate the formation of cracks in the ice that are particularly important for Weddell seals as breathing points (Nicol, Worby, Strutton, & Trull, 2006). Heerah et al. (2013) demonstrated that mCDW was the main water mass used by the Weddell seals in winter at DDU. However, at Davis, direct evidence of seals exploring the mCDW is not available as the tags deployed for this study did not record both salinity and temperature.

The fact that seal hunting dives were performed over shallower bathymetry instead of the available deeper areas also suggests that these shallower areas could facilitate prey accessibility and capture. Moreover, Plötz, Bornemann, Knust, Schröder, and Bester (2001) suggested that a hunting seal descending from the surface would not switch to benthic foraging as long as *P. antarcticum* were available in the upper water column. Seals foraging in shallower areas could switch easily to benthic prey if their initial prey targets became depleted in the water column.

## 5 Conclusion

Our study highlighted some of the key foraging strategies adopted by Weddell seals during the Antarctic winter. At both sites, Weddell seals remained in coastal areas associated with dense sea‐ice over shallow bathymetry that are surrounded by deep canyons and depressions. In these areas, Weddell seals concentrated their foraging activity within the range of their breathing abilities, likely until prey depletion, before moving to another area. The differences observed in distances traveled, foraging activity, and diving behavior resulted from differences in sea‐ice conditions and prey targeted between the focal sites. Overall, Weddell seal foraging behavior responded to the physical aspects of their environment (seafloor topography and sea‐ice features) that are likely to be associated with better prey availability and accessibility as well as reliable access to breathing sites. At finer scales, the foraging behavior of Weddell seals likely responded to the distribution and availability of prey in the water column, switching from pelagic to benthic foraging, exhibiting diurnal behavior, and complex diving behaviors.

Despite similar foraging strategies and habitat use between and within the two focal sites, we observed high levels of interindividual variability, an adaptation that allows Weddell seals to respond to environmental perturbations (Chambert et al., 2012). However, such behavioral plasticity complicates quantifying the general impact, changes in climate, or the environment may have on Weddell seals. Indeed, it is possible that Weddell seal populations at different locations (e.g., Antarctic Peninsula, Weddell Sea, McMurdo Sound (Ainley, Larue, Stirling, Stammerjohn, & Siniff, 2015), DDU, and Davis) and individuals within each population may respond differently to changes in their environment.

## Conflict of Interest

None declared.

## Data Accessibility

All the datasets are available on the Integrated Marine Observing System (IMOS) website: https://imos.aodn.org.au/imos123/home.
