# Supplementary material for: Contrasting behavior between two populations of an ice‐obligate predator in East Antarctica
Source: Ecol Evol. 2016 Dec 20;7(2):606–18. doi: 10.1002/ece3.2652 (PMC5243189; doi:10.1002/ece3.2652)
Supplement: Supplementary file 1 [file ECE3-7-606-s001.docx]

**Supplementary information**


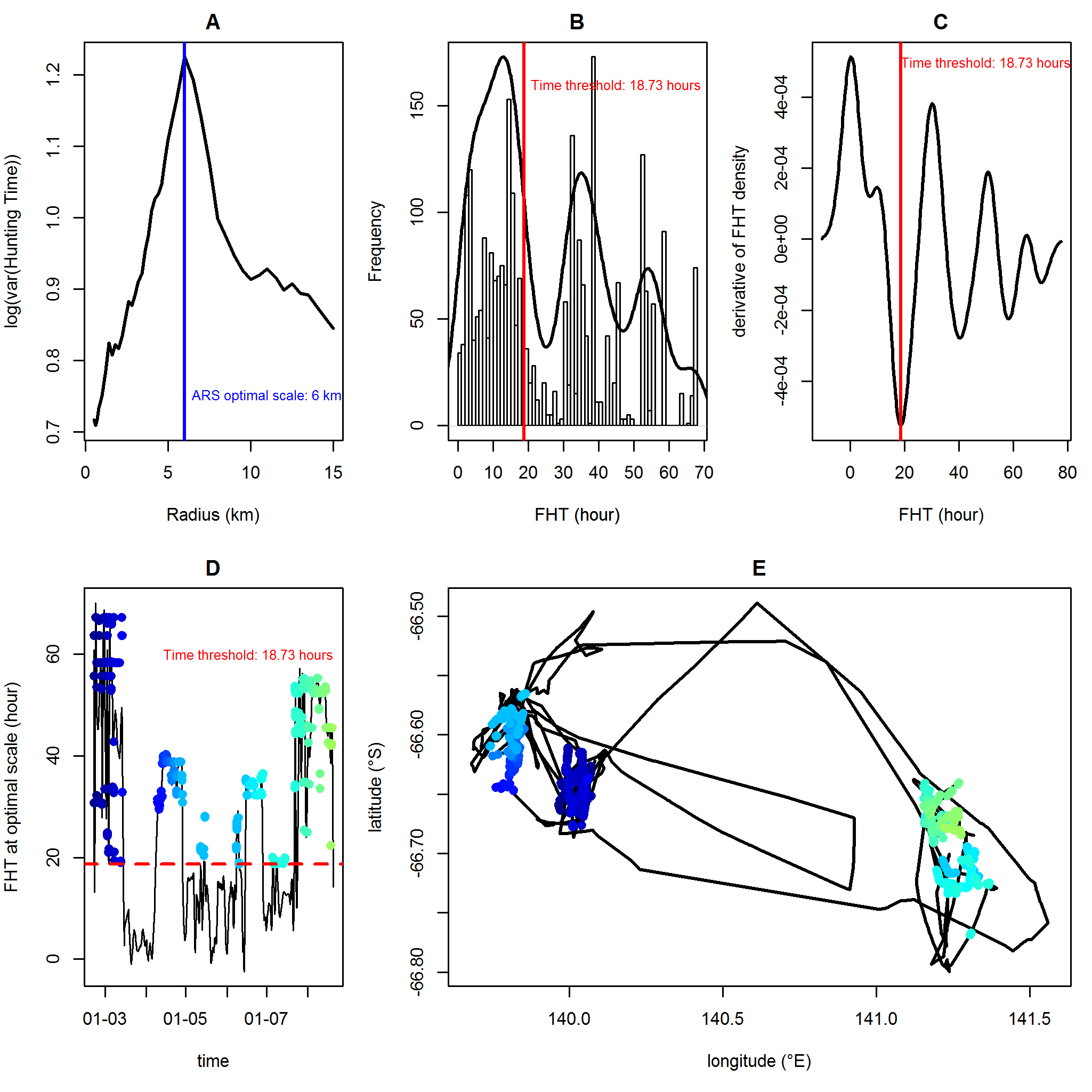


Figure S1. First hunting time procedure to identify ARS optimal scale and behavioural switch represented for one individual. A: Variance in First-Hunting Time (FHT) analysis as a function of circle radius for each individual. The maximum peak in variance indicates the scale of the most intensive search behaviour and is indicated by a blue line (FHT). B: Frequency distribution and density plot (black line) of FHT values. C: derivative of FHT values’ density. D: temporal evolution of FHT values. For (B), (C) and (D) the time threshold to discriminate transit vs hunting behaviour is indicated by a red line. E: map representing the track of one individual and associated behavioural mode. For (C) and (E) hunting dives are represented by coloured dots according to the day of year.


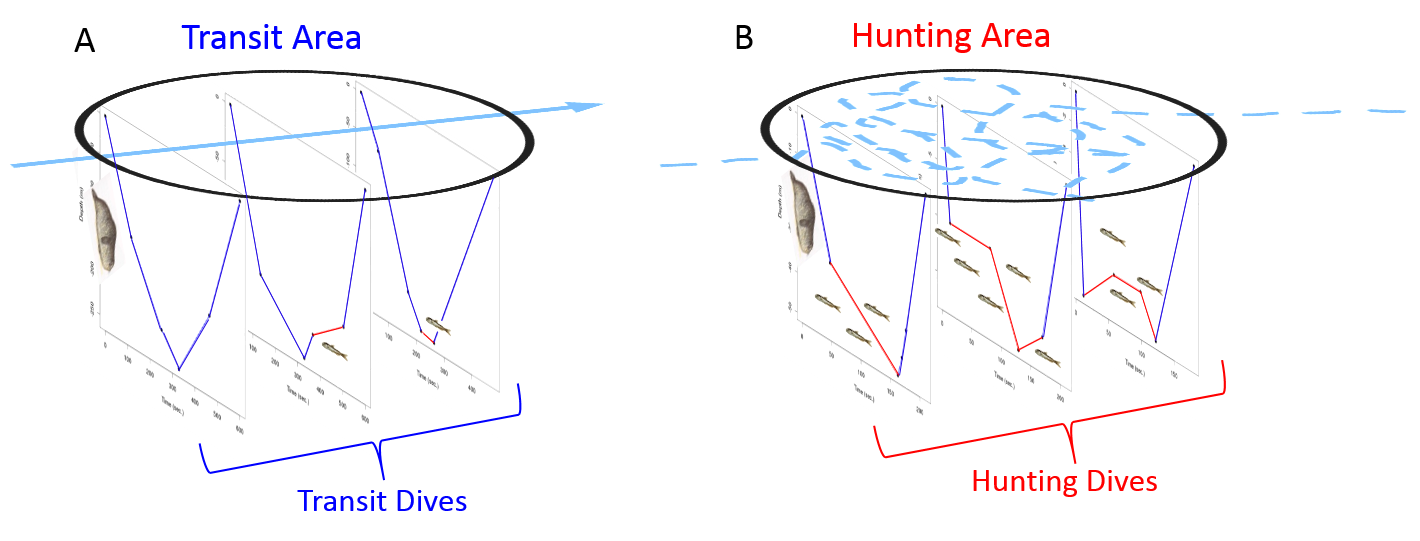


**Figure S2.** Illustration of the First Hunting Time method. This method adopts the same procedures used in First Passage Time analysis, except that instead of measuring the time required to cross a circle of given radius, it sums the total time spent hunting within that circle. This adaptation allowed us to discriminate hunting vs transit areas (at the optimal spatial scale for each individual), taking into account individuals’ horizontal and vertical behaviour (see Figure S1 and methods supplementary information “First Hunting Time analysis”).


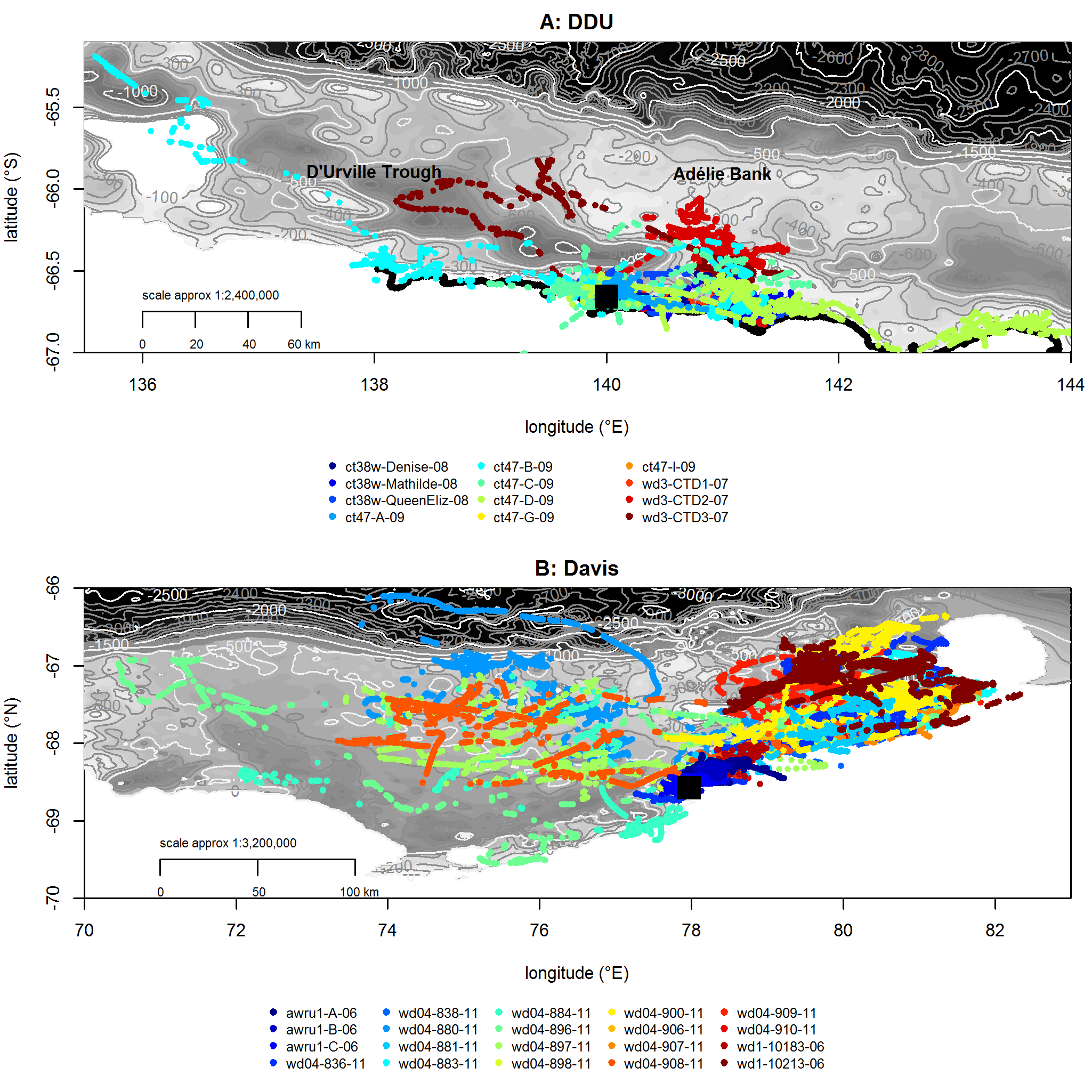


Figure S3. Tracks of SRDL equipped Weddell seals from Dumont d’Urville (A) and Davis (B) during 2007-09 and 2006-07/11 respectively. The tracks were corrected using a continuous random walk model (R package “crawl”). The deployment site at each colony is indicated by a black square.


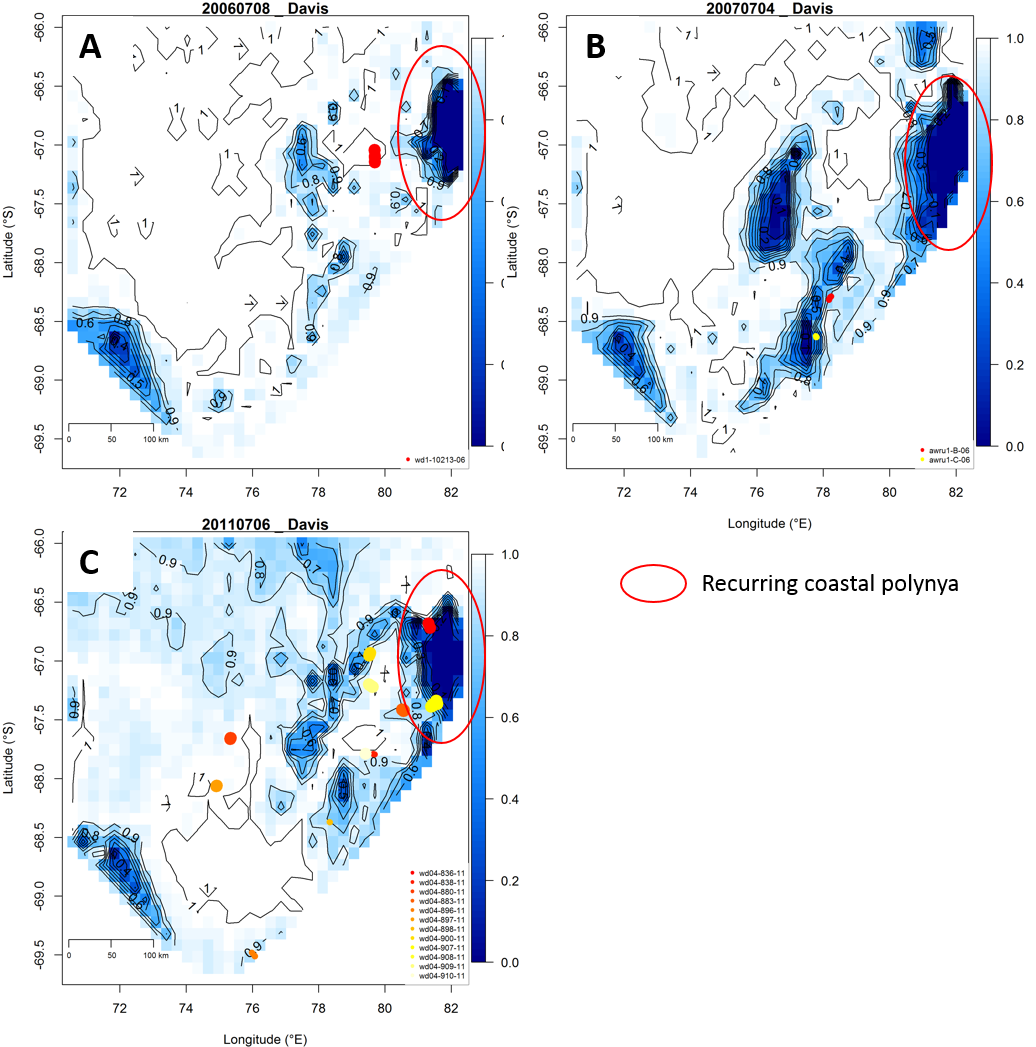


**Figure S4.** Daily maps of sea-ice concentration obtained from the AMSRE satellite for the Davis site. It shows the existence of a recurring small coastal polynya on a pluri-annual basis (2006: A, 2007: B, 2011: C) in winter (beginning of July for the images presented).

**Appendix S1. Environmental data**

Given that the bathymetry around the Antarctic is not equally well described we used two bathymetric datasets according to their spatial coverage. A fine-scale bathymetry dataset (Beaman *et al.* 2011, 100 m cell grid resolution) was merged with locations from 10 seals (out of 12) from DDU that did not travel west of 138°E, which was the longitudinal limit of this data set (http://data.aad.gov.au/). Broader-scale GEBCO bathymetry (30 sec [~1 km] cell grid resolution) was merged with seal locations from Davis and the two individuals from DDU that travelled west of 138°E (http://www.gebco.net/). The bathymetric slope (hereafter “slope”) was calculated for each grid cell from the bathymetry values of the eight neighbouring cells using the R software package *raster* ((R Development Core Team 2008); function *terrain*; (Hijmans 2014)). Finally, the 100 bathymetry and slope values associated with each possible dive location were averaged, giving a mean value and its standard deviation for each location along the main track.

Sea-ice concentration was sourced from AMSR-E daily sea-ice concentration images (http://www.iup.physik.uni-bremen.de:8084/amsr/amsre.html). Each pixel in the image (5.95 km x 6.57 km) had an ice concentration value (ranging from 0 to 100 %). Sea-ice concentration values were extracted for each dive following the same procedure outlined by the bathymetry and slope extraction method above. Two other variables were calculated using the sea-ice concentration images: (i) the distance to the closest area of ice concentrations below 20 % (hereafter “distance to ice edge”) instead of the 15 % threshold commonly used because DDU and Davis are located in coastal fast-ice areas (M. Vancopenolle, pers. com.) and; (ii) an index of the spatial variation of sea-ice concentration in the vicinity of each dive. The latter was calculated as the standard deviation of sea-ice concentrations within a 25 km radius of each dive (named “sdice25”).

**Appendix S2. First Hunting Time analysis**

First Hunting Time analysis was performed using a customized R algorithm (available upon request to the authors) We tested radii from 500 m to 15 km; increasing by 100 m increments between 500 m and 1 km, by 200 m increments between 1.2 km and 5 km, and by 500 m increments between 5.5 km and 15 km. Radii were chosen to reflect the small scale movements of Weddell seals and to be ecologically meaningful for this species (*e.g.* sea-ice concentration is expected to constrain seal habitat selection as they rely on ice-holes to breath). The minimum radius size was not less than 500 m because only 25 % of dive locations were associated with estimated error lower than 500 m. The spatial scale of concentrated search effort was defined, for each seal, as the mean peak in log-transformed variance in FHT (to make the variance independent of the magnitude) relative to radius size (Fig. S1a). We chose to retain the optimal spatial scale for each seal instead of averaging among all individuals, as their range of displacements varied dramatically from one another.

(Fauchald & Tveraa 2003) defined search areas as the areas associated with the longest FPT. Similar to Thums *et al.* (2011), we used the distribution of FHT density estimates to find a time threshold discriminating the mode of lower FHT values (*i.e.* “transit”) from all other higher modes (*i.e.* “hunting”) (see details in supplementary material and Fig. S1). A simple approach was used to find this time threshold automatically for each individual. First, it identified the FHT value corresponding to the first maximum of the density estimates curve (*i.e.* lower FHT values mode Fig. S1b): FHTdens_max_. Second, it uses the derivative of the density estimates curve (Fig. S1c) to calculate the first inflexion point after FHTdens_max,_ (*i.e.* inflexion point of the lower FHT value mode, Fig. S1 b-c). Dives with FHT values below the time threshold were defined as “transit” dives, whereas dives with FHT values above the time threshold were considered “hunting” dives.

**Appendix S3. Statistical analysis**

We fitted a series of generalized mixed effect models (GLMM) with multivariate normal random effects, using penalized quasi-likelihood (R package “MASS”, function “glmmPQL”, Venables and Ripley 2002). We used this type of GLMM instead of the one more commonly used (*i.e.* provided by the R package “lme4”, Bates et al 2014; *e.g.* (O’Toole *et al.* 2014) because it enables the addition of an auto-correlation term to avoid violation of the “independence” assumption when dealing with time series dataset (*e.g.* tracking data, dive series, environmental time series) (Zuur *et al.* 2009; Zuur, Ieno & Elphick 2010). Missing and outlier values were removed leaving 62188 dives (73% of the retained dives). Non-collinearity was verified between continuous variables using Pearson correlation (coef < 0.5) and the variance inflection factor (VIF) (Zuur *et al.* 2010). All explanatory variables were standardized (centred and scaled) to facilitate model convergence and enable comparison of their respective contribution (using their corresponding slope coefficients). Due to computational limitations, models were performed using a random sub-sample of dives (1/3 dives). We started with a full model that included all environmental variables and meaningful variable interactions (*i.e.* influence of site). We then implemented a stepwise procedure to remove non-significant variables with the threshold set at p-value < 0.05 (Zuur *et al.* 2009). Finally, GLMMs were validated by examining the residuals distribution and checking for any potential trend between residuals and each explanatory variable (*i.e.* verification of homogeneity) (Zuur *et al.* 2009).

**References for supplementary information**

Beaman, R.J., O’Brien, P.E., Post, A.L. & De Santis, L. (2011) A new high-resolution bathymetry model for the Terre Adélie and George V continental margin, East Antarctica. *Antarctic Science*, **23**, 95–103.

Fauchald, P. & Tveraa, T. (2003) Using first-passage time in the analysis of area-restricted search and habitat selection. *Ecology*, **84**, 282–288.

Hijmans, R.J. (2014) raster: Geographic data analysis and modeling. *R package version 2.2-5*.

O’Toole, M., Hindell, M., Charrassin, J. & Guinet, C. (2014) Foraging behaviour of southern elephant seals over the Kerguelen Plateau. *Marine Ecology Progress Series*, **502**, 281–294.

R Development Core Team. (2008) *R: A Language and Environment for Statistical Computing*. R Foundation for Statistical Computing, Vienna, Austria.

Thums, M., Bradshaw, C.J. & Hindell, M.A. (2011) In situ measures of foraging success and prey encounter reveal marine habitat-dependent search strategies. *Ecology*, **92**, 1258–1270.

Zuur, A.F., Ieno, E.N. & Elphick, C.S. (2010) A protocol for data exploration to avoid common statistical problems: Data exploration. *Methods in Ecology and Evolution*, **1**, 3–14.

Zuur, A., Ieno, E.N., Walker, N., Saveliev, A.A. & Smith, G.M. (2009) *Mixed Effects Models and Extensions in Ecology with R*. Springer.
